# Investigating the Role of β-Disodium Glycerophosphate and Urea in Promoting Growth of *Streptococcus thermophilus* from Omics-Integrated Genome-Scale Models

**DOI:** 10.3390/foods13071006

**Published:** 2024-03-26

**Authors:** Chengjie Hou, Xin Song, Zhiqiang Xiong, Guangqiang Wang, Yongjun Xia, Lianzhong Ai

**Affiliations:** School of Health Science and Engineering, University of Shanghai for Science and Technology, Shanghai 200093, China; houcj2001@163.com (C.H.); daohongxuan@126.com (X.S.); xiongzhiq@163.com (Z.X.); 1015wanggq@163.com (G.W.); dreamup@126.com (Y.X.)

**Keywords:** *Streptococcus thermophilus*, transcriptomic, metabolomic, genome-scale metabolic network model, growth mechanism

## Abstract

This study investigates the impact of urea and β-GP on the growth of *Streptococcus thermophilus* S-3, a bacterium commonly used in industrial fermentation processes. Through a series of growth experiments, transcriptome, metabolome, and omics-based analyses, the research demonstrates that both urea and β-GP can enhance the biomass of *S. thermophilus*, with urea showing a more significant effect. The optimal urea concentration for growth was determined to be 3 g/L in M17 medium. The study also highlights the metabolic pathways influenced by urea and β-GP, particularly the galactose metabolism pathway, which is crucial for cell growth when lactose is the substrate. The integration of omics data into the genome-scale metabolic model of *S. thermophilus*, iCH502, allowed for a more accurate prediction of metabolic fluxes and growth rates. The study concludes that urea can serve as a viable substitute for β-GP in the cultivation of *S. thermophilus*, offering potential cost and efficiency benefits in industrial fermentation processes. The findings are supported by validation experiments with 11 additional strains of *S. thermophilus*, which showed increased biomass in UM17 medium.

## 1. Introduction

*Streptococcus thermophilus*, as a significant commercial fermentation starter, is extensively utilized in products such as yogurt and cheese, with its consumption being substantial and its market value exceeding 40 billion USD [1]. The high-density fermentation of *S. thermophilus* has a crucial impact on industrial applications. M17 medium, a formula widely used for the laboratory culture of *S. thermophilus*, contains β-disodium glycerophosphate (β-GP: 19 g/L) as its highest proportion component. β-GP is also the most expensive component (the price of the analytical reagent-grade one is approximately 200~650 USD/500 g), which greatly limits the industrial application of M17 medium. The purpose of adding β-GP to M17 medium is to replace phosphate buffer, as phosphate forms precipitates with alkali metal ions. Therefore, the role of β-GP in the medium is often attributed to its buffering capacity [2,3], but there is no definitive research to confirm this speculation. Subsequent studies found that β-GP inhibits the growth of *Lactobacillus bulgaricus* and *Lactobacillus helveticus* [3]. Recent studies on β-GP also suggest that it may have antimicrobial properties by binding to bacterial cell membranes to induce cell lysis [4]. Therefore, while β-GP improves the performance of the medium, it may also have potential negative effects. In order to develop a medium more suitable for industrial production, it is necessary to conduct in-depth research on the role of β-GP in the growth of *S. thermophilus*.

The proliferative effect of urea on *S. thermophilus* has been confirmed in our previous studies, where the addition of urea to a chemically defined medium (CDM) significantly increased the biomass of *S. thermophilus* S-3 [5]. This is beneficial for the high-density cultivation of *S. thermophilus*. Research indicates that the urease activity is significantly correlated with lactose utilization [6]. Studies on energetically discharged cells with blocked transcription and translation suggested that the production of ammonia can stimulate glycolysis [7]. We also found that the addition of urea to the CDM could maintain the pH of the fermentation broth under neutral conditions, which gave us an insight: perhaps urea can replace β-GP in M17 medium for the cultivation of urease-positive bacteria. By analyzing the similarities and differences between β-GP and urea in promoting the growth of *S. thermophilus*, regulatory targets for the proliferation of urease-negative bacteria can be screened.

Cell growth metabolic regulation is one of the most complex systems, and the application of systems biology methods can globally establish the relationship between genes, transcription, and metabolism. Omics technology, as a rapidly developing new technology, plays a huge role in various forms of biological research. Single omics data can only reflect differences in the part of the regulatory scale, so the joint analysis of multi-omics can more comprehensively explain biological mechanisms. Many methods have been developed for the integration of transcriptomics and metabolomics, but these methods mainly rely on subjective judgment when interpreting biological associations [8]. Therefore, people hope to integrate omics data under the background of biological knowledge associated with organisms. Genome-scale metabolic network models (GEMs) are a systems biology tool constructed based on the genome, which can be used to place the interactions of different biological components in context and interpret these networks [9]. Integrating omics data into GEMs allows for the study of organisms in a specific context, exploring metabolic mechanisms from a systems biology perspective. These methods have been successfully applied in model organisms such as *E. coli* [10] and yeast [11]. REMI (Relative Expression and Metabolomic Integrations) is the first method that can integrate both transcriptomic and metabolomic data into GEMs for analysis [12]. This approach can better reflect different physiological states of cells than original models.

To deeply understand the mechanisms by which β-GP and urea promote the proliferation of *S. thermophilus*, we measured the physiological phenotypes, transcriptomes, and metabolomes of *S. thermophilus* under different culture conditions, and used REMI to integrate these data into GEM for analysis. By establishing condition-specific models for different culture conditions, we elucidated the metabolic status of the cells and predicted the metabolic flux within the cells. This study provides a basis for improving the culture medium of *S. thermophilus* by replacing β-GP with urea, and lays the foundation for subsequent research on the high-density culture and regulatory targets of *S. thermophilus*.

## 2. Materials and Methods

### 2.1. Micro-Organisms and Culture Conditions

The *S. thermophilus* strains used in this study were all preserved in a −80 °C refrigerator in our laboratory. The strains were activated by culturing on M17 solid medium (containing 20 g/L lactose, the same below) at 37 °C for 24 h. A representative single clone colony was picked and inoculated into M17 liquid medium to continue culturing until the exponential phase. After obtaining the cells by centrifugation (5000× *g*, 5 min), they were washed three times with sterile physiological saline. The cells were then resuspended and inoculated (inoculation amount: 2%) into three types of media: M17 medium (M17), M17 medium with urea replacing β-GP (UM17), and M17 medium without β-GP (M17-β). They are cultured at 37 °C for 12 h, and samples are taken regularly to measure various indicators.

### 2.2. Determination of Sugar, Lactate, Urea, and Biomass

The concentrations of lactose, galactose, and lactic acid were analyzed using a high-performance liquid chromatography system (model 2695; Waters Corp., Milford, MA, USA). The concentrations of urea were determined using the diacetylmonoxime method. Cell dry weight was determined by weighing. All methods were based on previously established methods [5].

### 2.3. Transcriptomic Analysis

When bacterial cells have grown to the late exponential phase, the fermentation broth was centrifuged (5000× *g*, 5 min) to collect the cells. The cells were washed three times with pre-cooled PBS (0.01 M), and then resuspended in PBS to the same OD_600_ value (1.0 ± 0.05). The suspension was quantitatively aliquoted into new 1.5 mL centrifuge tubes. After centrifugation, the supernatant was discarded, and the cells were flash-frozen in liquid nitrogen and stored in a −80 °C refrigerator. Total RNA was extracted from the tissue using CTAB method and genomic DNA was removed. Only RNA samples of high quality were employed for the construction of the sequencing library. Depletion of ribosomal RNA (rRNA) was carried out using the RiboCop rRNA Depletion Kit for Mixed Bacterial Samples (lexogen, Hampshire, MA, USA), as opposed to poly(A) purification. Following this, all mRNA was fragmented into short segments (200 nt) by the addition of fragmentation buffer. Double-stranded cDNA was then synthesized using random hexamer primers (Illumina, San Diego, CA, USA). During the synthesis of the second strand of cDNA, dUTP was incorporated in lieu of dTTP. The synthesized cDNA underwent end-repair, phosphorylation, and ‘A’ base addition as per Illumina’s library construction protocol. The RNA-seq transcriptome library was prepared following the Illumina^®^ Stranded mRNA Prep, Ligation (San Diego, CA, USA) using total RNA. The paired-end RNA-seq library was sequenced with the Illumina Novaseq 6000 (Illumina Inc., San Diego, CA, USA). The processing of original images to sequences, base-calling, and quality value calculations were performed. The clean reads were obtained by removing low-quality sequences, reads with more than 10% of N bases (unknown bases), and reads containing adaptor sequences. Three biological replicates were set up for each group. The transcription analysis was carried out by Shanghai Meiji Biomedical Technology Co., Ltd. (Shanghai, China), including sample preprocessing, RNA extraction, sequencing library construction, Illumina sequencing. Transcriptomic data are available from the Sequence Read Archive (SRA) repository of National Center for Biotechnology Information (accession number: PRJNA1081653).

### 2.4. Untargeted LC-MS/MS Metabolomic Analysis

For untargeted metabolomics analysis, six biological replicates were set up for each group. The method of bacterial cell collection was the same as transcriptome. A quantity of 50 mg cells was introduced into a 2 mL centrifuge tube, along with a grinding bead of 6 mm diameter. Metabolite extraction was performed using 400 μL of an extraction solution (methanol: water in a 4:1 *v/v* ratio) that contained an internal standard (L-2-chlorophenylalanine) at a concentration of 0.02 mg/mL. The samples were ground using the Wonbio-96c frozen tissue grinder (manufactured by Shanghai Wanbo Biotechnology Co., Ltd., Shanghai, China) for a duration of 6 min at −10 °C and 50 Hz. This was followed by low-temperature ultrasonic extraction for 30 min at 5 °C and 40 kHz. The samples were then left at −20 °C for 30 min, after which they were centrifuged for 15 min at 4 °C and 13,000× *g*. The supernatant was transferred to the injection vial for LC-MS/MS analysis. The LC-MS/MS analysis was conducted on a Thermo UHPLC-Q Exactive HF-X system equipped with an ACQUITY HSS T3 column (100 mm × 2.1 mm i.d., 1.8 μm; Waters, Milford, CT, USA) at Majorbio Bio-Pharm Technology Co. Ltd. (Shanghai, China). The mobile phases consisted of 0.1% formic acid in water: acetonitrile (95:5, *v*/*v*) (solvent A) and 0.1% formic acid in acetonitrile: isopropanol: water (47.5:47.5, *v*/*v*) (solvent B). The flow rate was set at 0.40 mL/min and the column temperature was maintained at 40 °C. The mass spectrometric data were collected using a Thermo UHPLC-Q Exactive HF-X Mass Spectrometer (Thermo Fisher, Waltham, MA, USA) equipped with an electrospray ionization (ESI) source operating in both positive and negative modes. The optimal conditions were set as follows: source temperature at 425 °C; sheath gas flow rate at 50 arb; Aux gas flow rate at 13 arb; ion-spray voltage floating (ISVF) at −3500 V in negative mode and 3500 V in positive mode, respectively; and normalized collision energy, 20–40–60 V rolling for MS/MS. Full MS resolution was 60,000, and MS/MS resolution was 7500. Data acquisition was performed with the Data-Dependent Acquisition (DDA) mode. The detection was carried out over a mass range of 70–1050 m/z.

### 2.5. Data Processing and Statistical Analysis

#### 2.5.1. Transcriptomics

The data generated from the Illumina platform were utilized for bioinformatics analysis. All analyses were conducted using the Majorbio Cloud Platform, a free online platform provided by Shanghai Majorbio Bio-pharm Technology Co., Ltd. The primary software and parameters are as follows: (i) Mapping reads to reference genome: High-quality reads from each sample were mapped to the customer-provided reference genome [13]. (ii) rRNA contamination assessment: In this step, 10,000 raw reads randomly selected from each sample were aligned to the Rfam database using the blast method. The percentage of rRNA in each sample was calculated based on the annotation results, providing an estimate of rRNA contamination. (iii) Expression analysis: Gene and isoform abundances were quantified from RNA-Seq data using RSEM [14]. RSEM computes maximum likelihood abundance estimates using the Expectation Maximization algorithm for its statistical model. This includes the modeling of paired-end and variable-length reads, fragment length distributions, and quality scores to determine which transcripts are isoforms of the same gene. The transcripts per million reads (TPM) method was used to calculate expression levels. (iv) Differential expression analysis: Differentially expressed genes (DEGs) were identified for each dataset using DESeq2 [15]. (v) Gene Ontology (GO) enrichment analysis: The Gene Ontology project provides an ontology of defined terms representing gene properties, covering three domains: Cellular Component, Molecular Function, and Biological Process. GO enrichment analysis identifies GO terms that DEGs are enriched in, helping to illustrate the differences between two particular samples at functional levels. The Goatools [16] is used to identify statistically significantly enriched GO terms using Fisher’s exact test. FDR correction is performed to reduce Type-1 error by the BH method. After multiple testing corrections, GO terms with an adjusted *p*-value ≤ 0.05 are considered significantly enriched in DEGs. (vi) KEGG enrichment analysis: Differentially expressed genes typically interact with each other in vivo to perform certain biological functions. Compared with the whole genome background, KEGG enrichment analysis can identify the most important biological metabolic pathways and signal transduction pathways that DEGs are involved in KOBAS 2.0 [17].

#### 2.5.2. Metabolomics

The LC/MS raw data were preprocessed using Progenesis QI 2.0 software (Waters Corporation, Milford, CT, USA), resulting in the export of a three-dimensional data matrix in CSV format. This matrix contained sample information, metabolite names, and mass spectral response intensities. The data matrix was cleaned by removing internal standard peaks, known false-positive peaks (including noise, column bleed, and derivatized reagent peaks), and then de-duplicated and peak pooled. Metabolites were identified by searching databases, primarily HMDB 5.0 [18], Metlin [19], and Majorbio Database [20]. The resulting data matrix was uploaded to the Majorbio cloud platform for further analysis. The data matrix was preprocessed by retaining at least 80% of the metabolic features detected in any set of samples. For specific samples with metabolite levels below the lower limit of quantification, the minimum metabolite value was estimated, and each metabolic signature was normalized to the sum. To mitigate errors caused by sample preparation and instrument instability, the response intensities of the sample mass spectrometry peaks were normalized using the sum normalization method. Variables from QC samples with a relative standard deviation (RSD) greater than 30% were excluded, and the remaining data were log10-transformed to obtain the final data matrix for subsequent analysis. The R package “ropls” (Version 1.6.2) was used to perform principal component analysis (PCA) and orthogonal least partial squares discriminant analysis (OPLS-DA), with a 7-cycle interactive validation to evaluate the stability of the model. Metabolites with a Variable Importance in the Projection (VIP) greater than 1 and a *p*-value less than 0.05 were determined to be significantly different metabolites. These were based on the VIP obtained by the OPLS-DA model and the *p*-value generated by Student’s t-test. Differential metabolites between two groups were mapped into their biochemical pathways through metabolic enrichment and pathway analysis based on the KEGG database [21]. These metabolites could be classified according to the pathways they were involved in or the functions they performed. Enrichment analysis was used to analyze whether a group of metabolites appears in a function node or not. The principle was that the annotation analysis of a single metabolite develops into an annotation analysis of a group of metabolites. The Python package “scipy.stats” was used to perform enrichment analysis to obtain the most relevant biological pathways for experimental treatments.

### 2.6. Genome-Scale Model Refinement

Based on the previous model iCH492, we further refined the model and performed gap filling based on transcriptome and metabolome data. In order to study the impact of β-GP on cell growth, reactions related to β-GP metabolism were added.

### 2.7. Integration of Transcriptome Data and Metabolome Data into Genome-Scale Models

The REMI was used to integrate transcriptome and metabolome data into the model. REMI can combine gene expression data and metabolomics data as constraints for the model, greatly reducing the feasible flux solution space. Moreover, REMI extensively enumerated alternative solution spaces. Due to the complexity of the metabolic network, there may be multiple feasible pathways leading to the same phenotype, so the results provided by REMI can more accurately reflect the physiological state of the cell. REMI represents the ratio of gene expression/metabolic abundance under two conditions as the flux perturbation of each reaction and imposes it as a constraint on individual fluxes. It can integrate the model in three ways: (1) REMI-Gex: integrating transcriptome data into GEM; (2) REMI-M: integrating metabolome data into GEM; and (3) REMI-GexM: integrating both transcriptome and metabolome data as model constraints. The transcriptome and metabolomics data of M17-β vs. M17 and M17-β vs. UM17 were used to impose constraints on the model, and the calculation of metabolic flux of substrates and products refers to the previously established method. Analyzing the model according to the method of N. Hadadi et al. [22], the upper and lower bounds of the specific growth rate of the context-specific models are constrained to within ± 0.1 of the actual value based on the phenotypic experiment.

REMI aims to maximize the consistency between gene expression, metabolite concentration, and metabolic flux levels for a given condition pair. By formulating an optimization problem, the number of constraints imposed by relative gene expression and metabolite abundance is maximized. These constraints are integrated into the model to predict growth phenotypes. Two scores are calculated: Theoretical Maximum Consistency Score (TMCS), which represents the number of available omics data; and Maximum Consistency Score (MCS), which represents the number of omics data consistent with metabolic flux, i.e., the number of genes/metabolites that can be integrated into the model. The MILP formula can enumerate alternative sets from given constraints. The IBM CPLEX 12.8 solver is used to calculate the maximum consistency score. All programming source codes used to analyze the data were available at GitHub (https://github.com/houcj2001/GP, accessed on 20 February 2024).

## 3. Results

### 3.1. Growth Characteristics of S. thermophilus S-3 under Different Conditions

**Figure 1:**
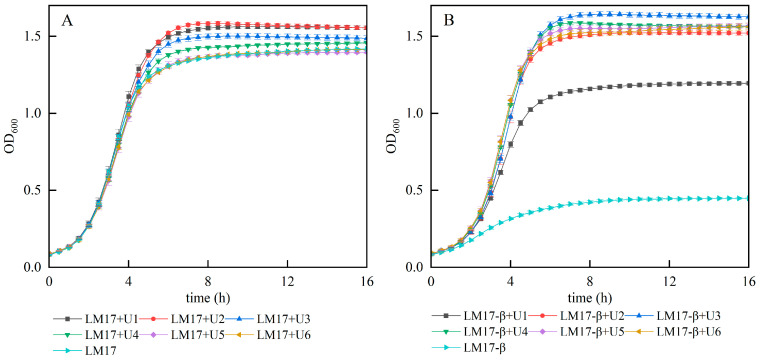
Effect of urea addition on the biomass of S. thermophilus S-3. (**A**) The growth curve of S-3 in M17 medium with different concentrations of urea. (**B**) The growth curve of S-3 in M17-β medium with different concentrations of urea.

Our prior research has substantiated that urea can enhance the growth of *S. thermophilus* S-3 and augment its biomass in CDM medium. To further investigate whether urea can increase the biomass of *S. thermophilus* S-3 in M17 medium, we conducted growth experiments by adding different concentrations of urea (1~6 g/L, denoted as U1~U6) to both M17 medium and β-GP-deficient M17 medium (M17-β). The experimental results (Figure 1) indicated that a lower concentration of urea (1~2 g/L) in M17 medium is more conducive to cell growth than a higher concentration, with the maximum biomass observed at a urea concentration of 2 g/L (Figure 1A). In experiments where urea replaced β-GP (Figure 1B), the cell biomass in the β-GP-deficient medium was minimal, but a low concentration of urea (1 g/L) could significantly enhance the biomass. When the amount of urea added reached 3 g/L, the biomass peaked and was significantly higher than that in M17 medium (*p* < 0.05), suggesting that urea can substitute for β-GP in M17 medium. However, in both M17 and M17-β media, when the urea concentration exceeded a certain threshold, the biomass showed a certain degree of decline, implying that an excessively high concentration of urea is not conducive to the increase in biomass. Therefore, the optimal level of urea addition was determined to be 3 g/L.

**Figure 2:**
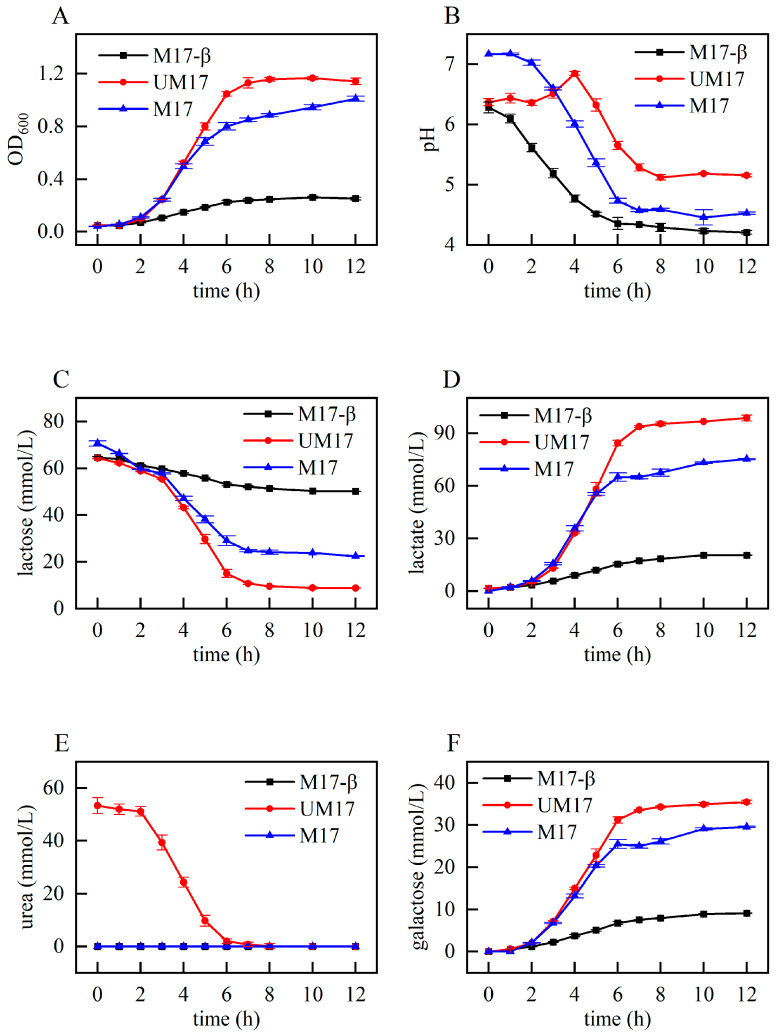
Fermentation characteristics of *S. thermophilus* under different conditions: (**A**) OD_600_; (**B**) pH; (**C**) the concentration of lactose; (**D**) the concentration of lactic acid; (**E**) the concentration of urea; and (**F**) the concentration of galactose.

In order to further assess the impact of urea and β-GP on the growth of *S. thermophilus* S-3 and provide phenotypic data for subsequent analysis, we examined the fermentation characteristics of *S. thermophilus* S-3 under three conditions: M17-β, M17, and UM17 (urea: 3 g/L). As depicted in Figure 2A, the growth cycles (lag phase, logarithmic phase, and stationary phase) of the three groups were essentially consistent. The UM17 group exhibited the largest biomass, while the biomass of the M17-β group was significantly lower than the other two groups. The consumption of lactose and the production of lactic acid and galactose (Figure 2C,D,F) were all in line with the trend of biomass changes. The M17-β group and the M17 group demonstrated similar trends of pH decline, but the final pH of the M17-β group was lower than that of the M17 group. The pH of the UM17 group, however, showed a trend of initially rising, and then falling, which is consistent with the phenomenon observed in the CDM [5].

### 3.2. Transcriptome Analysis

**Figure 3:**
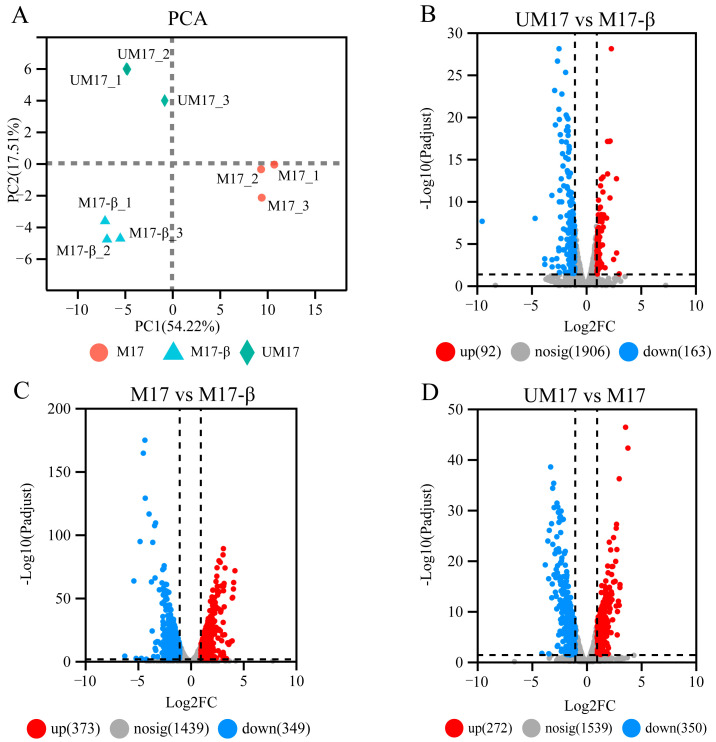
Transcriptional profiling of *S. thermophilus* S-3 under different conditions. (**A**) PCA scoring plot. The points represented biological replicates. (**B**–**D**) Volcano plot of differential genes. The red, blue, and gray dots represented upregulated, downregulated, and non-significant difference genes, respectively.

To compare differences in mRNA levels caused by urea and β-GP, gene expression profiles in *S. thermophilus* S-3 were analyzed by transcriptomic analysis based on RNA-seq. The principal component analysis (PCA) result (Figure 3A) showed that changes in gene expression were induced by intergroup difference rather than intra-group difference, which meant that urea and β-GP all changed the gene expression abundance of *S. thermophilus* S-3 under fermentation. Differential expression analysis showed that 622 DEGs were identified in UM17 compared to M17, of which 272 and 350 genes were upregulated and downregulated, respectively (Figure 3D; see also Table S1C). Relative to M17-β, 722 DEGs were identified in M17 and only 255 DEGs were detected in UM17 (Figure 3B,C; see also Table S1).

**Figure 4:**
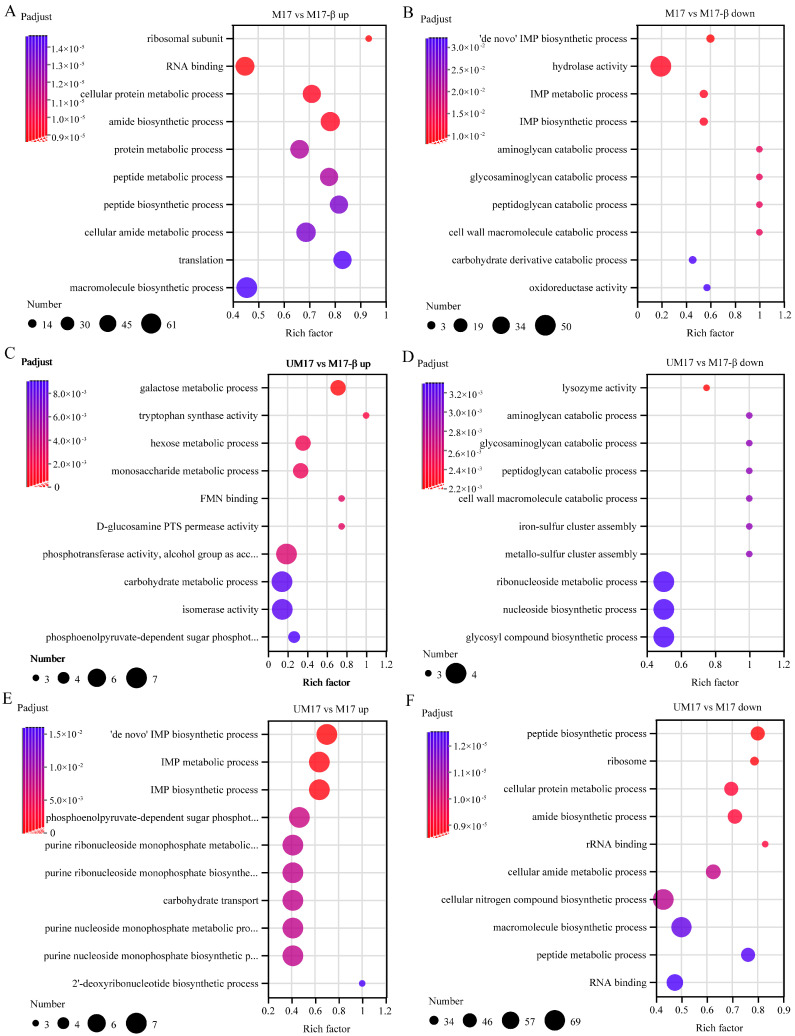
The top 10 upregulated and downregulated enriched GO annotations of M17 vs. M17-β (**A**,**B**), UM17 vs. M17-β (**C**,**D**), UM17 vs. M17 (**E**,**F**). The *x*-axis represents the Rich factor, the *y*-axis represents the GO term, the size of the dot indicates the number of genes/transcripts in this GO term, and the color of the dot corresponds to different ranges of FDR (*p*-value corrected).

KEGG enrichment analysis (Figure S2) and GO enrichment analysis (Figure 4) were performed on DEGs to evaluate the biological functions of DEGs caused by urea and β-GP. The top 10 upregulated and downregulated GO enrichment results were shown in Figure 4A–F. Compared to M17-β and M17, the upregulated genes in the UM17 group were enriched in carbohydrate transport and the phosphoenolpyruvate-dependent sugar phosphotransferase system (Figure 4C,E; see also Table S2). Relative to M17-β and UM17, the upregulated genes in M17 were enriched in the peptide biosynthetic process, cellular protein metabolism, and the macromolecule biosynthetic process (Figure 4A,F; see also Table S2), while the downregulated genes were mainly enriched in the IMP biosynthetic process (Figure 4B,E). By comparing M17 vs. M17-β and M17 vs. UM17, it can be found that the two sets of differential gene sets have many common DEGs (Figure S1). Among the top 30 upregulated and downregulated DEGs, 10 common genes were found in each, of which three upregulated DEGs were related to tryptophan synthase, and one upregulated DEG was related to glycerol uptake permease (Table S3).

### 3.3. Metabolome Analysis

**Figure 5:**
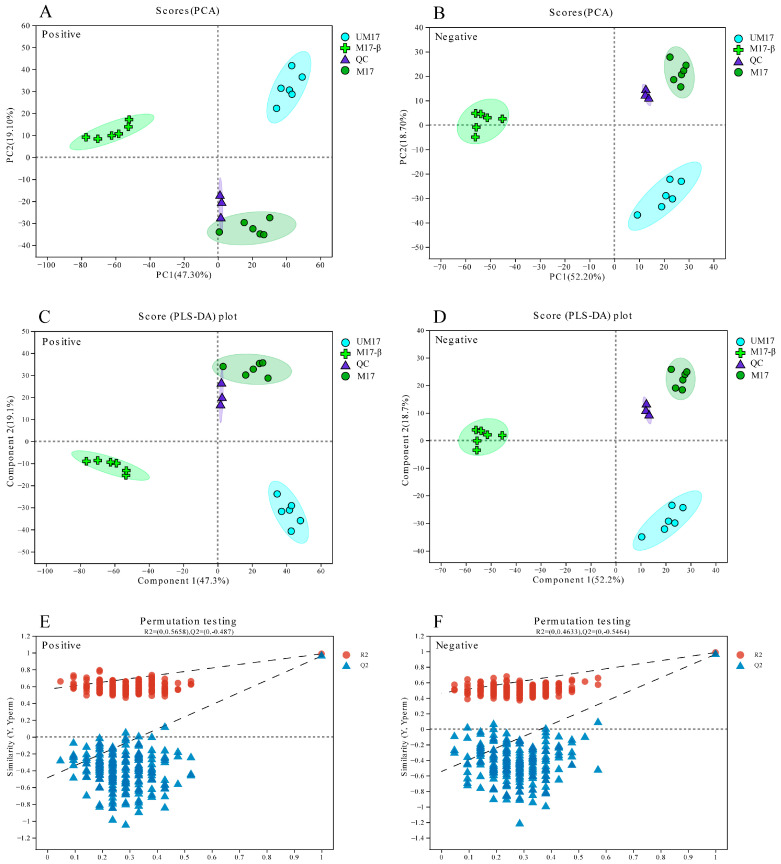
Metabolic profiling of *S. thermophilus* S-3 under different conditions. (**A**,**B**) PCA scoring plot. (**C**,**D**) PLS-DA scoring plot. The quality control (QC) represented a mixture of all samples. (**E**,**F**) PLS-DA model validation. The *x*-axis represents the permutation retention of the permutation test, the *y*-axis represents the values of the R2 (blue dots) and Q2 (red triangles) permutation tests, and the two dashed lines represent the regression lines of R2 and Q2, respectively.

**Figure 6:**
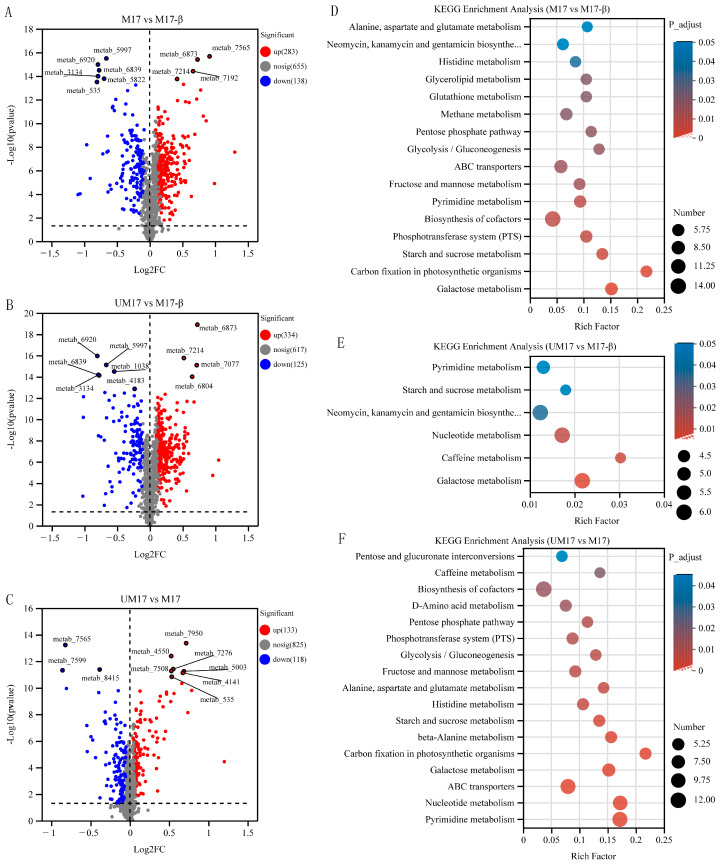
Volcano plot and KEGG enrichment analysis of intracellular metabolites. (**A**–**C**) Volcano plot of M17 vs. M17-β, UM17 vs. M17-β, and UM17 vs. M17. The *x*-axis represents the fold change in metabolite expression between the two groups, i.e., log2FC, and the *y*-axis represents the statistical test value of the difference in metabolite expression, i.e., −log10(*p*-value) value. The higher the value, the more significant the expression difference. (**D**–**F**) KEGG enriched pathways of M17 vs. M17-β, UM17 vs. M17-β, and UM17 vs. M17. The *x*-axis represents the Rich factor, and the *y*-axis represents the KEGG pathway. The size of the bubbles represents the number of compounds enriched in the metabolic set in that pathway, and the color of the bubbles indicates the size of the *p*-value for different enrichment significances.

Metabolic variations among comparisons of UM17 vs. M17-β, M17 vs. M17-β, and UM17 vs. M17 were performed using LC-MS. A total of 1076 annotated metabolites were detected (number of ESI+ and ESI− ions was 583 and 493, respectively; Table S4). PCA and PLS-DA showed significant differences among all groups in both positive and negative models (Figure 5A–D). The intercept of the Q2 regression line obtained by the Permutation test is less than 0.05 (Figure 5E,F), indicating that the model robustness is reliable and no overfitting has occurred. Based on the variable importance in projection (VIP) score >1 and *p*-values < 0.05, relative to M17-β, 459 and 421 DAMs were screened in UM17 and M17, respectively (Figure 6A,B). Notably, just 251 annotated metabolites were screened between UM17 and M17 (Figure 6C).

Thirty, forty, and forty-six metabolites had VIP scores > 2 in UM17 vs. M17-β, M17 vs. M17-β, and UM17 vs. M17, respectively (Table S5). Among the metabolites with VIP scores > 2.0, twenty-two appeared in both UM17 vs. M17-β and M17 vs. M17-β, e.g., Isofebrifugine, Sphingosine, Avocadene, and 4-Methylumbelliferone. Compared to M17-β, both UM17 and M17 had fewer upregulated DAMs than downregulated DAMs (Table S5). However, in UM17 vs. M17, the number of upregulated DAMs was roughly equal to the number of downregulated DAMs. Compared to UM17 and M17-β, M17 had 13 identical DAMs, and these DAMs showed the same upregulation and downregulation patterns (see Table S6), suggesting that the abundance changes of these metabolites are likely caused by β-GP. The upregulated DAMs mainly appeared in the carbohydrate metabolism pathway, while the downregulated DAMs were mostly oligosaccharide compounds. The KEGG pathway enrichment analysis was performed among each group (Figure 6D,F). Galactose metabolism, purine metabolism, pyrimidine metabolism, starch and sucrose metabolism, and glyceride metabolism were all significantly enriched in the three analysis groups (UM17 vs. M17-β, M17 vs. M17-β, and UM17 vs. M17-β).

### 3.4. Omics-Based Curation and Omics Integration into GEM

#### 3.4.1. GEM Curation

Based on transcriptomic and metabolomic data, we have refined the existing GEM of *S. thermophilus*, iCH492. By re-annotating the genome of *S. thermophilus* S-3, we have supplemented and corrected some gene–protein–reaction (GPR) relationships in the model. Our goal was to investigate the potential role of β-GP in cellular metabolism, so we added reactions related to β-GP metabolism into the GEM. In the M17 group, the expression level of the gene *GLPF*, which encodes the glycerol uptake permease, was significantly higher than that in the M17-β and UM17 groups. Considering the absence of detectable glycerol in the medium and cells, we hypothesize that glycerol uptake permease may be involved in the transport of β-GP due to their structural similarity. The expression level of the gene *suhB* was also significantly higher in the M17 group compared to the M17-β group. The *suhB* gene encodes glycerol-2-phosphate phosphatase, which catalyzes the conversion of β-GP to glycerol and phosphoric acid (reaction G2PP). We also observed elevated expression levels of the six genes comprising the phosphoric acid permease gene cluster in the M17 group, leading us to include the G2PP reaction in the model. The iterated model is named iCH502.

#### 3.4.2. GEM Curation

REMI was used to integrate the relative gene expression and metabolite abundance data from M17-β vs. M17 and M17-β vs. UM17 into iCH502, respectively. In REMI, relative gene expression and metabolite abundance data can be integrated into the model separately (REMI-Gex or REMI-M), or simultaneously (REMI-GexM). REMI-GexM can integrate experimental data as constraints to better reflect the metabolic mechanisms within the cell; hence, we utilized the REMI-GexM model for analysis.

Based on phenotypic experimental data, we generated three models, iCH502-M17-β, iCH502-M17, and iCH502-UM17, for REMI analysis. The TMCS and MCS of M17-β vs. M17 were 132 and 72, respectively, while those of M17-β vs. UM17 were 45 and 23, respectively. An important feature of REMI is its ability to generate all alternative solutions consistent with the growth phenotype. In this study, we used alternative solutions to study the impact of β-GP and urea on the distribution of metabolic fluxes within cells. REMI analysis showed that there were 18 alternative solutions in the M17-β vs. UM17 group, and 920 alternative solutions in the M17-β vs. M17 group. The predicted growth rates obtained via REMI-GexM were consistently higher for both the M17 and UM17 groups compared to the M17-β group (Table 1). These results outperformed those obtained from Flux Balance Analysis (FBA). Furthermore, the model predicted a positive reaction flux for F_0_F_1_-ATPase in the UM17 group, suggesting that this reaction can indeed generate ATP—a finding consistent with our previous conclusions on urea metabolism [5]. Although the prediction accuracy of REMI has significantly improved compared to FBA, there is still a deviation between the predicted value and the experimental value. To improve this deviation and better reflect the growth phenotype, we constrained the model’s growth rate in subsequent analyses (see Section 2).

**Figure 7:**
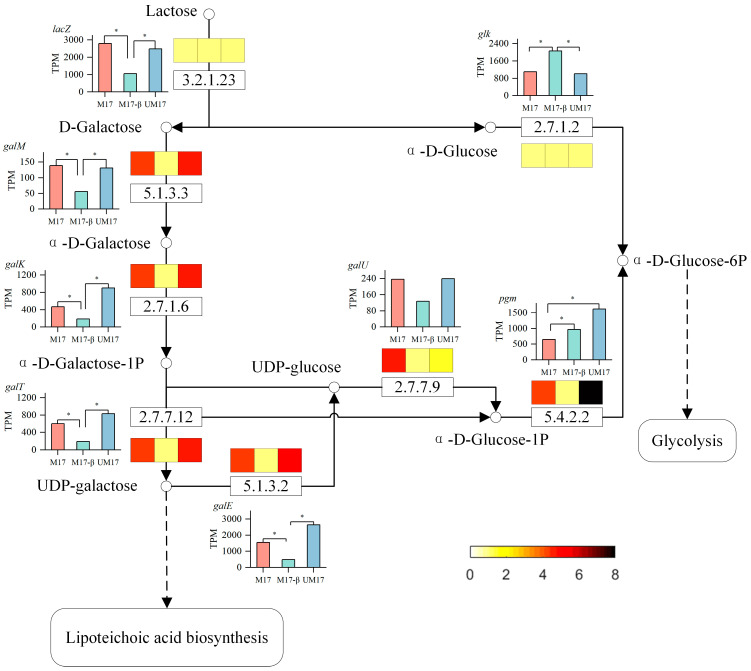
Schematic diagram of galactose metabolism flow. The bar graph indicates the expression of the gene corresponding to the reaction, and * indicates a significant difference between groups. The color-blocked graphs indicate the reaction fluxes calculated by REMI (data have been normalized), color from yellow to black corresponds to a flux range of 0 to 8, with darker colors indicating higher flux levels.

Using the KEGG mapper tool [23], all common reactions generated by REMI in all alternative solutions were mapped onto the KEGG pathway. These common reactions play a crucial role—they persist across all alternative solutions, regardless of the metabolic network’s adaptations to varying physiological conditions. As such, they serve as potential targets for metabolic regulation. The list of the common reactions is proved in Table S8. Nucleotide metabolism is the pathway with the most common reactions in both cases, with 13 reactions (out of a total of 31 reactions) in M17 and 8 reactions (out of a total of 20 reactions) in UM17, most of which are related to DNA synthesis. The next is carbohydrate metabolism, where multiple reactions in galactose metabolism appear simultaneously in both the M17 and UM17 groups, suggesting that the galactose metabolism pathway may be related to the proliferation of *S. thermophilus*. To delve deeper, we further analyzed the flux of galactose metabolism based on the model and omics data (Figure 7). When lactose serves as the carbon source, the enzyme β-galactosidase (3.2.1.23) hydrolyzes lactose into galactose and glucose. Glucose directly enters the glycolysis pathway. Part of galactose is excreted outside the cell through the lactose transport system. Another part of galactose enters the galactose metabolism pathway. It is catalyzed by aldose-1-epimerase (5.1.3.3) and galactokinase (2.7.1.6) to produce α-D-Galactose-1-phosphate, which reacts with UDP-glucose to produce UDP-galactose and α-D-Glucose-1-phosphate. Unlike other bacteria, the glucose moiety in the teichoic acid repeating unit structure of the *Streptococcus* genus is replaced by a galactose moiety [24], so one of the main metabolic fluxes of UDP-galactose is to enter the lipoteichoic acid synthesis pathway. α-D-Glucose-1-phosphate re-enters the glycolysis pathway. The relative abundance of UDP-galactose in the M17 and UM17 groups is downregulated compared to the M17-β group, and the relative abundance of α-D-Glucose-1-phosphate in the M17 group is upregulated compared to the other two groups.

### 3.5. Validation of UM17 Medium Universality in Urease-Positive S. thermophilus

**Figure 8:**
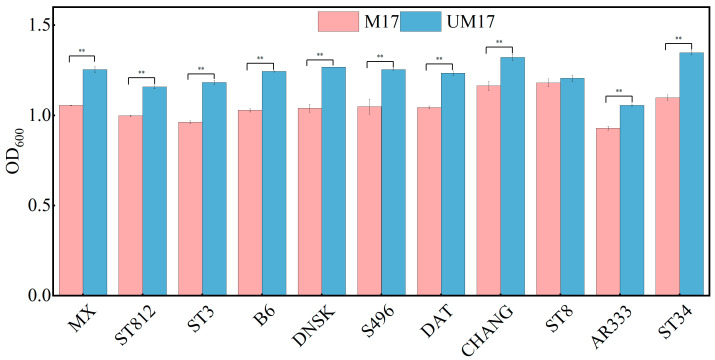
Growth validation experiment of *S. thermophilus* in UM17 medium. ** indicates extremely significant (*p* < 0.01).

Through omics analysis, we observed significant transcriptional differences between the M17 and UM17 groups. However, both groups exhibited several common enriched metabolic pathways in the metabolome analysis. Additionally, REMI analysis revealed that both M17 and UM17 groups stimulated the flux of the galactose metabolic pathway, providing increased biomass precursors for cell growth. This suggests that the transcriptional mechanisms promoting cell growth via β-GP and urea may differ, but there is substantial metabolic overlap, with urea showing potential as a substitute for β-GP. To verify the applicability of UM17 medium for *S. thermophilus* cultivation, we cultured an additional 11 strains of this bacterium from our laboratory collection. After 12 h of cultivation, 10 out of 11 bacterial strains exhibited significantly higher biomass in UM17 medium compared to M17 medium, with only one strain showing no statistically significant difference between the two culture conditions (Figure 8).

## 4. Discussion

This study attempted to explain the impact of β-GP and urea on the proliferation of *S. thermophilus*. By integrating omics data into GEM, additional constraints can be applied to the model, which can more accurately reflect the distribution of metabolic fluxes in cells. For this study, we manually curated and iteratively refined the GEM of *S. thermophilus* S-3 based on omics data, resulting in the model iCH502. Phenotypic experiments, and transcriptomic and metabolomic analyses revealed significant differences in *S. thermophilus* under different culture conditions, but the molecular mechanisms causing these differences have not yet been revealed. The results of the fermentation experiment indicate that both urea and β-GP have a positive effect on the growth of *S. thermophilus* S-3, with urea demonstrating superior performance. In previous research, we used FBA to evaluate the role of urea metabolism in the growth of *S. thermophilus*. FBA is performed based on the maximization of an objective function (such as biomass yield). However, this method does not reflect the impact of environmental stress and other factors on cells. Based on a small amount of substrate and product quantitative data, the specific growth rates calculated by FBA were all higher than the experimental values (Table 1). However, the integration of omics data using REMI significantly improved the consistency between the predicted values and the experimental values, demonstrating that the integration of omics data can enhance the predictive capability of the model. REMI-generated alternative solutions allow us to characterize the intracellular state of *S. thermophilus* by identifying the changing fluxes in metabolic pathways.

Transcriptomic analysis showed that the gene for glycerol uptake permease in the M17 group was significantly upregulated, leading us to speculate that, in addition to transporting glycerol, it may also be involved in the transport of β-GP. During the simulation analysis of the model, although we set a high uptake rate for the β-GP transport reaction (10 mmol/gDW/h), the flux of this reaction was small in all alternative solutions (0.38 ± 0.14 mmol/gDW/h). This suggests that, while β-GP can be utilized by cells as a substrate, it is not directly metabolized in large amounts. After hydrolysis, β-GP provides phosphate ions to the cells. As is well-known, phosphate groups play an important role in energy metabolism. The lack of phosphates in the M17-β group medium may be one of the reasons for its smaller biomass.

During the growth process of *S. thermophilus*, a large amount of lactic acid was produced and excreted to the outside of the cell, which gradually increases the osmotic pressure outside the cell. In addition, the large amount of lactic acid produced by the cells during the exponential phase cannot be excreted in time, leading to a decrease in the intracellular pH. This decrease mainly occurs because the H^+^/lactic acid symport performs poorly under high external lactic acid concentrations [25]. Batch fermentation experiments showed that the pH of both the M17-β group and the M17 group have significantly decreased. Considering that the lactic acid production of the M17 group is several times that of the M17-β group (Figure 2D), but the final pH is still higher than M17-β group, it indicates that β-GP can play a certain buffering role. In previous research, we have confirmed that urea metabolism causes the consumption of H+ inside the cell, and F_0_F_1_-ATPase transports H^+^ from outside the cell to the inside, keeping the pH inside and outside the cell near neutral, and REMI results once again confirm our previous views [5]. The phosphate group contained in β-GP gives it a buffering capacity within a certain range, and the phosphoric acid obtained after β-GP hydrolysis can also play a certain buffering effect inside the cell. However, β-GP requires proton symport, and, when one molecule of β-GP is transported into the cell, two protons will enter the cell, which will cause a decrease in the pH inside the cell, to some extent offsetting the buffering effect of phosphoric acid, so the buffering effect of β-GP should mainly be reflected outside the cell. From the perspective of maintaining the cytoplasmic pH close to neutral, urea has a better effect than β-GP. On the other hand, the proton gradient potential provided by urea metabolism allows extracellular protons to enter the cell through the F_0_F_1_-ATPase channel, producing a large amount of ATP for cell growth, so, when culturing urease-positive *S. thermophilus*, urea can completely replace β-GP and have a better effect.

In the M17 group, three upregulated DEGs are related to tryptophan synthase. In previous research, we found that the absence of tryptophan has a detrimental effect on the growth of *S. thermophilus* [5], implying that genes related to tryptophan synthase are likely closely related to growth. The common reaction analysis also identified a reaction (IGPS) related to tryptophan metabolism, the product of which is used for the further synthesis of tryptophan. In addition to being used for protein synthesis, some studies suggest that tryptophan can be catalyzed by tryptophanase to form indole. As a signaling molecule, indole plays a role in responding to environmental stress and regulating biofilm formation [26].

In the common reaction analysis, both the M17 and UM17 groups have multiple reactions belonging to the galactose metabolism pathway. In the case of perfect stoichiometry, the theoretical galactose–lactose exchange coefficient should be 0.5 g galactose/g lactose, but, in reality, the majority of strains are below this value [27]. This implies that galactose is not completely excreted, but a portion is directly utilized by the cells. Compared to the M17-β group, the gene expression and reaction flux obtained by REMI in the galactose metabolic pathway in the M17 and UM17 groups were all upregulated, combined with the significant enrichment of the galactose metabolic pathway in the metabolome, all indicating that β-GP and urea can enhance the gene expression and metabolic flux of the galactose metabolic pathway. This suggests that galactose metabolism plays an important role in the growth of *S. thermophilus* when lactose is the substrate. Some key genes in galactose metabolism (such as *galM*, *galK*) are expected to be targets for subsequent modification. This method can be utilized for the selection of additional modification targets suitable for growth regulation, as shown in Table S8.

In *S. thermophilus*, galactose is used to synthesize teichoic acid, polysaccharides, and nucleoside sugars [28]. Metabolic flux shows that more UDP-galactose in the M17 and UM17 groups flows to the lipoteichoic acid synthesis pathway, and the results of transcriptome enrichment analysis and metabolome VIP analysis also show the activity of large-molecule synthesis such as peptidoglycan. The expression of genes *tagE* related to lipoteichoic acid synthesis in the M17 group is upregulated compared to the other two groups, implying that β-GP may play a role in promoting cell wall synthesis. It is worth noting that, although the reaction flux of UDP-glucose to α-D-Glucose-1-phosphate in the M17 group is higher than that in the UM17 group, the reaction flux from α-D-Glucose-1-phosphate to α-D-Glucose-6-phosphate is lower than that in the UM17 group. Metabolome data also show that the relative abundance of α-D-Glucose-1-phosphate in the M17 group is significantly higher than that in the UM17 group, which is consistent with the lower expression of the gene *pgm*. The difference between the UM17 group and the M17 group may be explained by the limitation of the total protein (enzyme) amount. In unicellular organisms, the finite intracellular volume restricts the unlimited increase of enzyme molecules, thereby limiting the concentration of available enzymes [29]. The M17 group needs to synthesize more proteins in the cell wall synthesis pathway to resist acid stress, while the UM17 group avoids acid stress due to urea metabolism. The optimal growth of bacteria is a balance between maximizing yield and minimizing protein burden [30], thus the UM17 group can allocate more carbon for the synthesis of biomass precursors.

REMI yielded as many as 920 alternative solutions for the M17-β vs. M17 group, suggesting that β-GP broadly impacts cellular metabolic pathways. In the transcriptome enrichment analysis, the M17 group showed upregulation in the ribosomal pathway, indicating that the cell was actively synthesizing proteins, which may be related to the cell’s response to environmental stress [31]. Researchers found that the more easily metabolized α-isomer did not have an inhibitory effect in studies where β-GP inhibited the growth of certain lactic acid bacteria. In our study, we also found that changing to different brands of β-GP resulted in cellular growth inhibition (similar to M17-β); therefore, we hypothesize that the effect of β-GP on cell growth is related to its specific structure and plays a complex role in the cell’s response to acid stress.

Interestingly, all strains in our study tested positive for urease activity, suggesting that urease-positive strains are prevalent in *S. thermophilus*. Genomic analysis indicated that *S. thermophilus* S-3 possesses an 11-gene cluster encoding ureases, which is highly conserved within the species [32]. This cluster is considered part of the core genome of *S. thermophilus* [33]. Despite the existence of urease-negative mutants in industrial fermenters, UM17 medium remains practical due to the abundance of urease-positive strains and the cost-effectiveness of urea compared to β-GP, both in laboratory research and industrial production.

## 5. Conclusions

Our research has confirmed the effectiveness of substituting urea for β-GP in the cultivation of urease-positive *S. thermophilus*. The integration of transcriptome and metabolome data into the GEM analysis revealed that β-GP significantly alters intracellular metabolism. By acting as a buffer, promoting cell wall synthesis, and upregulating ribosomal expression, β-GP enhances the cell’s tolerance to acid stress. On the other hand, urea’s impact on cell proliferation primarily arises from the ammonia produced during metabolism. The binding of ammonia to protons maintains a neutral intracellular environment while also facilitating de novo ATP synthesis. Common reaction analysis indicates that nucleotide metabolism and galactose metabolism play crucial roles in *S. thermophilus*. Several reactions closely associated with cell growth were identified. These metabolic reactions hold promise as targets for regulating the growth metabolism of *S. thermophilus*, laying the foundation for high-density fermentation. In future studies, we aim to further investigate these identified targets using gene-editing and metabolic-engineering approaches.

## Figures and Tables

**Table 1:** Experimental and model prediction results.

	μ (h^−1^)			ATPase
	Experimental	FBA	REMI	
M17-β	0.284	0.88	0.104 ± 0.03 ^a^ 0.522 ± 0.099 ^b^	−0.655 ± 1.788 ^a^ −1.742 ± 2.021 ^b^
M17	0.519	0.87	0.494 ± 0.028	−1.329 ± 2.015
UM17	0.592	1.19	0.790 ± 0.250	6.267 ± 3.274

Experimental: Results from batch fermentation experiments; FBA: Optimization results from Flux Balance Analysis; REMI: Simulation results through REMI; ATPS3R: Metabolic flux of the F_0_F_1_-ATPase reaction: negative values indicate the reaction is proceeding in the direction of ATP consumption, while positive values indicate the reaction is proceeding in the direction of ATP production; ^a^: REMI results in M17-β vs. M17; ^b^: REMI results in M17-β vs. UM17.

## Data Availability

The original contributions presented in the study are included in the article/Supplementary Materials, further inquiries can be directed to the corresponding author.

## Supplementary Materials

The following supporting information can be downloaded at: https://www.mdpi.com/article/10.3390/foods13071006/s1, Figure S1: Venn plot of DEGs; Figure S2: KEGG enrichment analysis of DEGs, (*: *p* < 0.05, ***: *p* < 0.001); Table S1: DEGs between groups; Table S2: Transcriptome GO enrichment analysis; Table S3: Common DEGs in both M17 vs. UM17 and M17 vs. M17-β; Table S4: Results of LC-MS; Table S5: Metabolites of VIP scores; Table S6: Common DAMs in both M17 vs. UM17 and M17 vs. M17-β; Table S7: Added reactions in iCH502; Table S8: Results of common reaction analysis; Table S9: Model iCH502.

## Abbreviations

β-GP: β-disodium glycerophosphate; USD: US dollar; CDM: chemically defined medium; GEMs: genome-scale metabolic network models; M17: M17 medium; UM17: M17 medium with urea replacing β-GP; M17-β: M17 medium without β-GP; ESI: electrospray ionization; TPM: transcripts per million reads; DEGs: differentially expressed genes; GO: Gene Ontology; KEGG: Kyoto Encyclopedia of Genes and Genomes; RSD: standard deviation; PCA: principal component analysis; OPLS-DA: orthogonal least partial squares discriminant analysis; VIP: Variable Importance in the Projection; REMI: Relative Expression and Metabolomic Integrations; TMCS: Theoretical Maximum Consistency Score; MCS: Maximum Consistency Score; FDR: false discovery rate; FBA: Flux Balance Analysis; QC: quality control.
