# Supplementary figures and images for: Investigating the Role of β-Disodium Glycerophosphate and Urea in Promoting Growth of Streptococcus thermophilus from Omics-Integrated Genome-Scale Models
Source: Foods. 2024 Mar 26;13(7):1006. doi: 10.3390/foods13071006 (PMC11011449; doi:10.3390/foods13071006)

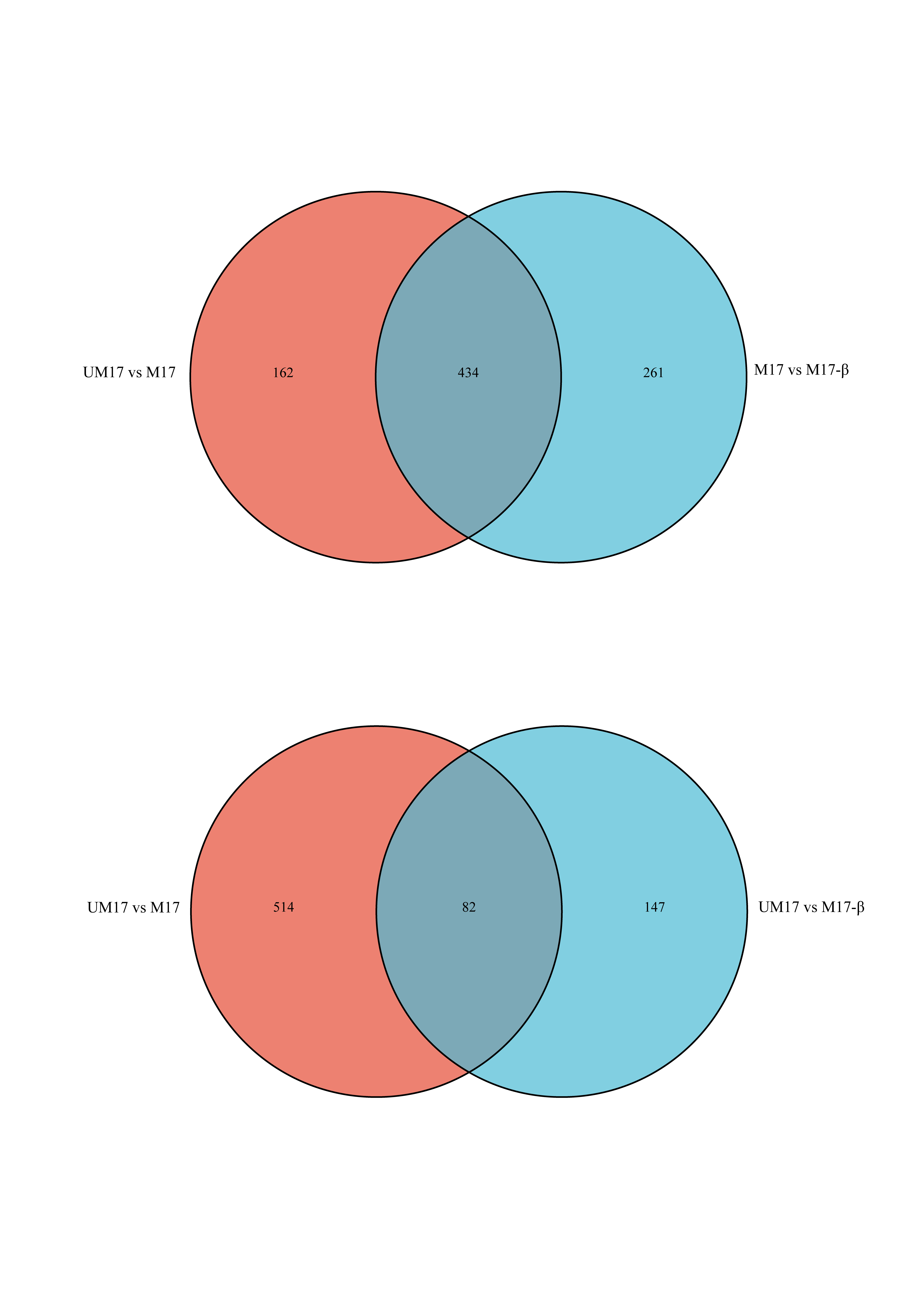

Supplement: Supplementary file 1 [file foods-13-01006-s001.zip › supplementary tables and figure/Fig. S1 Venn plot of DEGs.tif]

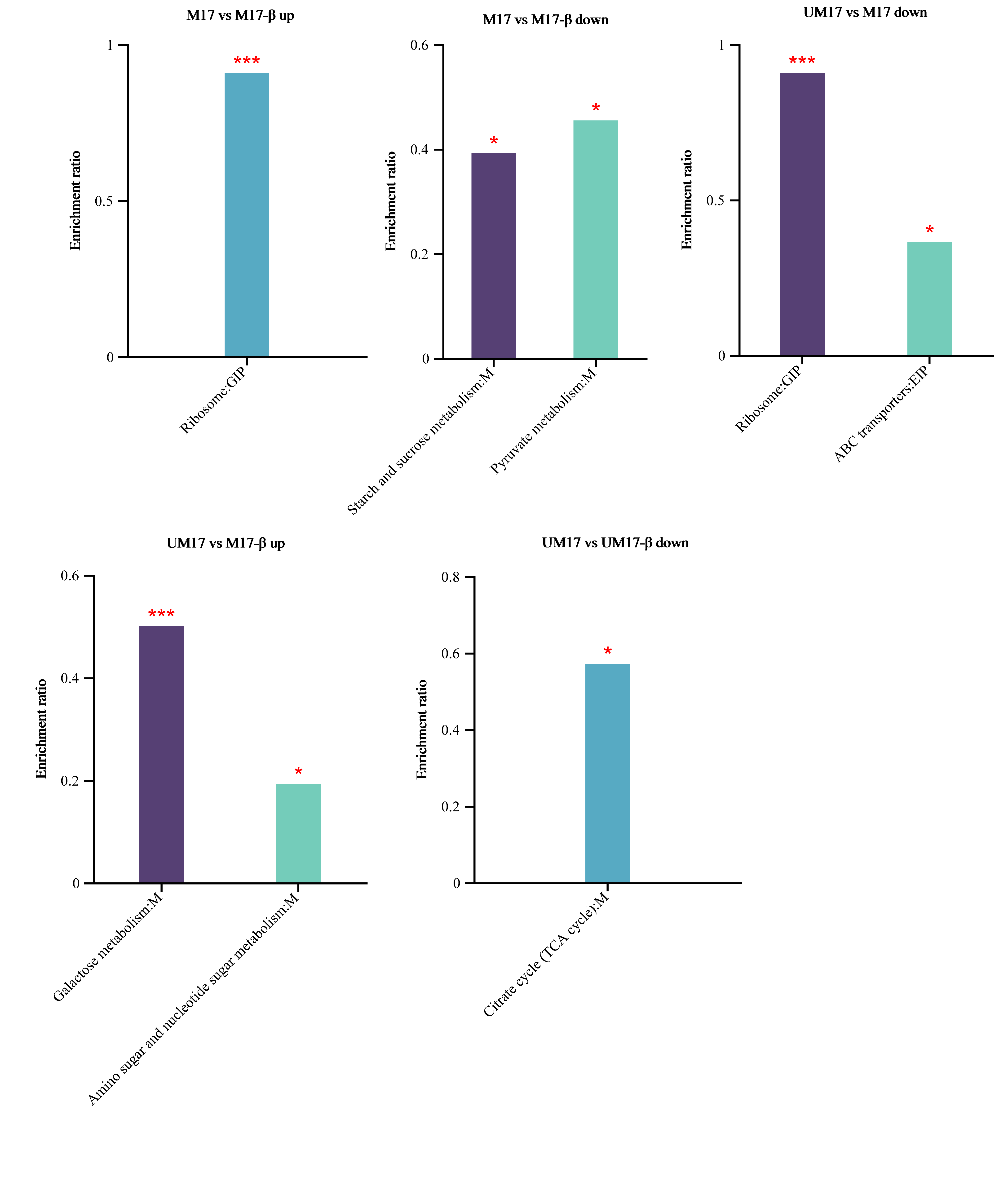

Supplement: Supplementary file 1 [file foods-13-01006-s001.zip › supplementary tables and figure/fig.S2 kegg enrichment of DEGs.tif]
